# Does hypoglycemia following a glucose challenge test identify a high risk pregnancy?

**DOI:** 10.1186/1742-4755-6-10

**Published:** 2009-07-14

**Authors:** Suzanne K Pugh, Dorota A Doherty, Everett F Magann, Suneet P Chauhan, James B Hill, John C Morrison

**Affiliations:** 1 Department of Obstetrics and Gynecology, Naval Medical Center Portsmouth, Portsmouth, VA, USA; 2 School of Women's and Infants' Health, University of Western Australia, Perth, Australia; 3 Department of Obstetrics and Gynecology, Aurora Health Care, West Allis, WI, USA; 4 Department of Obstetrics and Gynecology, University of Mississippi Medical Center, Jackson, MS, USA

## Abstract

**Objective:**

An association between maternal hypoglycemia during pregnancy with fetal growth restriction and overall perinatal mortality has been reported. In a retrospective pilot study we found that hypoglycemia was linked with a greater number of special care/neonatal intensive care unit admissions and approached significance in the number of women who developed preeclampsia. That study was limited by its retrospective design, a narrow patient population and the inability to perform multivariate analysis because of the limitations in the data points collected. This study was undertaken to compare the perinatal outcome in pregnancies with hyoglycemia following a glucose challenge test (GCT) to pregnancies with a normal GCT.

**Methods:**

Obstetric patients (not pre-gestational diabetics or gestational diabetes before 24 weeks were eligible. Women with a 1 hour glucose ≤ 88 mg/dL (4.8 m/mol) following a 50-gram oral GCT were matched with the next patient with a 1 hour glucose of 89–139 mg/dL. Pregnancy outcomes were evaluated.

**Results:**

Over 22 months, 436 hypoglycemic patients and 434 normal subjects were identified. Hypoglycemia was increased in women < 25 (p = 0.003) and with pre-existing medical conditions (p < 0.001). Hypoglycemia was decreased if pre-pregnancy BMI ≥ 30 (p = 0.008).

Preeclampsia/eclampsia was more common in hypoglycemic women. (OR = 3.13, 95% CI 1.51 – 6.51, p = 0.002) but not other intrapartum and perinatal outcomes.

**Conclusion:**

Hypoglycemic patients are younger, have reduced pre-pregnancy weight, lower BMIs, and are more likely to develop preeclampsia than normoglycemic women.

## Background

An association between maternal hypoglycemia during pregnancy with fetal growth restriction and overall perinatal mortality was reported in 1979 [1]. Since that time other investigators have used a variety of screening methods including both oral and intravenous glucose loading to identify hypoglycemia in pregnancy and relate this finding to pregnancy outcomes [2-9]. One of the most recent studies used the 100-gram oral glucose tolerance test and found a significantly lower incidence of both gestational diabetes and neonatal birth weights [10]. The best test to categorize pregnancies with hypoglycemia is uncertain, but the most widely available and frequently used test in most pregnancies would be the most effective screening test if correlated with pregnancy outcomes.

Previously, we undertook a retrospective pilot study to evaluate hypoglycemia following a 1 hour 50 g glucose challenge test (GCT) to determine if any association could be identified between hypoglycemia and an adverse pregnancy outcome [11]. That investigation found that hypoglycemia was linked with a greater number of special care/neonatal intensive care unit admissions and approached significance in the number of women who developed preeclampsia. The study was limited by its retrospective design, a narrow patient population and the inability to perform multivariate analysis because of the limitations in the data points collected.

The purpose of this prospective study was to compare the perinatal outcomes of pregnancies with hypoglycemia following a second trimester oral GCT to pregnancies with a normal GCT

## Methods

All pregnant women attending the Obstetric Clinics of the Naval Medical Center Portsmouth, Portsmouth, VA, and the Obstetric Clinics at the University of Mississippi Medical Center, Jackson, MS, who had not been diagnosed with pre-gestational diabetes or who had not been screened and identified as having diabetes prior to 24 weeks gestation were eligible for this study. Prospectively, these women routinely had a 1-hour 50-gram oral GCT as a screen for gestational diabetes performed at 24–28 weeks of gestational age. Hypoglycemia on the one hour oral GCT was defined for this investigation as it has been defined by others as a glucose level ≤ 88 mg/dl (4.8 m/mol) [1,11]. All women with a one hour glucose of ≤ 88 mg/dL (4.8 m/mol) (hypoglycemic group) were identified and normoglycemic controls the next patient with a one hour glucose following the glucose challenge test of 89 – 139 mg/dL (4.9–7.7 m/mol). Women with 1-hour glucose of ≥ 140 mg/dl (7.7 m/mol) following the 50-gram oral GCT would undergo a 3-hour glucose tolerance test with a 100-gram glucose load. A data sheet was completed and antenatal, intrapartum, and neonatal outcomes of the hypoglycemic group were compared with the normal group. Each patient was assigned a study number, and there were no patient identifiers on the data collection sheet.

This study was approved by the Chief, Navy Bureau of Medicine and Surgery, Washington, DC, through the local Clinical Investigation Program (Naval Medical Center – Portsmouth) (PO5-051). Approval was also obtained through the Investigation Review Board at the University of Mississippi Medical Center, Jackson, MS. The sharing of information between the 2 institutions was approved through the Navy Clinical and Cooperative Research and Development Agreement between the Naval Medical Center – Portsmouth and the University of Mississippi Medical Center (NMCP – 05–054).

We recruited 436 women with hypoglycemia and 434 women with normal blood glucose on the GCT to detect odds ratios ≥ 2.50 with 80% power for association between hypoglycemia and adverse pregnancy outcomes such as IUGR and preeclampsia when using multivariable logistic regression analysis.

Data were summarized using means and standard deviations or medians and interquartile ranges (IQR) for continuous data, depending on data normality. Frequency distributions were used to summarize categorical data. Univariate comparisons between women with hypoglycemia and normal glycemic controls were based on Mann-Whitney tests for continuous outcomes and Chi-square tests for categorical outcomes. Supplementary analyses utilized logistic regression modeling to investigate factors simultaneously associated with each pregnancy outcome considered such as preeclampsia, preterm delivery and IUGR. Odds ratios (OR) and 95% confidence intervals (CI) were used to summarize the covariate effects on the outcomes of interest. Relative risks have been obtained using the group prevalence of each outcome considered and OR estimates from the logistic regression analysis [12]. Statistical analysis was conducted using SPSS statistical software (SPSS, Inc., Version 11.0, Chicago, Illinois). *P*-values < 0.05 were considered statistically significant.

## Results

Eight hundred seventy women were recruited (436 and 434 with GCT ≤ 88 mg/dL (4.8 m/mol) and GCT range 89–139 mg/dL (4.9–7.7 m/mol) respectively) into the study between September 2004 and May 2006. Groups were similar in race, gravidity, parity and pregnancy weight gain (Table 1). Compared to women with normal GCT, hypoglycemic women were younger (24.7 vs. 25.9 years of age, p < 0.001), had lower pre-pregnancy weight (150 lb vs. 157 lb, p = 0.003) and lower pre-pregnancy BMI (27.0 vs. 26.5, p = 0.002). Higher rates of pre-existing medical conditions (12% vs. 5%, p < 0.001), prenatal complications (18% vs. 10%, p < 0.001) and antepartum hospitalizations (12% vs. 6%, p = 0.002) were found in hypoglycemic women (Table 1). Higher proportion of hypoglycemic women required induction of labor (30% vs. 20%, p < 0.0010) for a variety of reasons including rupture of the membranes without labor, non-reassuring antenatal testing, oligohydramnios, preeclampsia, intrauterine growth restriction, and preterm premature rupture of the membranes, Table 2). However, no differences in frequency of cervical ripening (p = 0.792), modes of delivery (p = 0.364) and reasons for cesarean delivery (p = 0.572) were found. Although the median gestational ages at delivery did not significantly differ between the groups, preterm deliveries were more frequent in the hypoglycemic group (p = 0.019).

**Table 1:** Maternal Characteristics

	Groups		
	Hypoglycemic (n = 436)	Controls (n = 434)	*P*-value

Maternal age^(1)^	24.7 ± 5.4	25.9 ± 5.5	0.001
			
Race			
Caucasian	179 (41%)	204 (47%)	
African American	214 (49%)	187 (43%)	
Other	38 (9%)	38 (9%)	0.179
			
Primigravidas	149 (34%)	149 (34%)	0.941
Nulliparous	190 (44%)	200 (46%)	0.421
Height (in)^(2)^	65 (63 – 67)	64 (63 – 67)	0.931
Pre-pregnancy weight (lb)^(2)^	150 (129 – 180)	157 (135 – 190)	0.003
Pre-pregnancy BMI ^(2)^	24.7 (21.8 – 29.5)	26.5 (22.9 – 32.3)	0.002
BMI			
<19	22 (7%)	15 (5%)	0.056
19–30	232 (69%)	209 (63%)	
≥30	82 (24%	107 (32%)	
			
Pregnancy weight gain (lb)^(2)^	33.0 (22.0–43.0)	32.0 (20.0 – 43.5)	0.393
History of spontaneous preterm birth	30 (7%)	23 (5%)	0.330
Any pre-existing medical conditions	54 (12%)	23 (5%)	<0.001
Chronic hypertension^(3)^	42 (78%)	19 (83%)	0.442
Other conditions^(3,4)^	12 (22%)	4 (17%)	0.764
			
Any prenatal complications	79 (18%)	44 (10%)	<0.001
Preeclampsia/eclampsia	35 (8%)	10 (2%)	<0.001
Preterm Labor	19 (4%)	15 (4%)	0.600
Oligohydramnios	11 (3%)	1 (0.2%)	0.006
Unexplained elevation of MSFAP	4 (1%)	7 (2%)	0.384
Other prenatal complications^(5)^	19 (4%)	10 (2%)	0.130
			
Antepartum hospitalization	53 (12%)	26 (6%)	0.002
Duration of stay (days)^(2,3)^	3 (3 – 7)	4 (3 – 4)	0.284

(1) mean ± standard deviation shown; (2) medians and interquartile ranges (1st – 3rd quartile) are shown; (3) percentages for subsets of patients with condition present are shown; (4) other conditions include gestational diabetes (n = 3), sickle cell disease (n = 3), multiple sclerosis (n = 3), PPROM (n = 1), lupus APA syndrome (n = 1), protein C deficiency (n = 1), renal disease (n = 1), seizure disorder (n = 1), ulcerative colitis (n = 1) and FV Leiden (n = 1); (5) GHTN (n = 7), PPROM (n = 4), gestational diabetes (n = 4), placenta praevia (n = 3), pyelonephritis (n = 3), 2 vessel cord (n = 3), DVT (n = 1), PE (n = 1), cholestasis (n = 1), cerclage (n = 1) and gastroschisis (n = 1).

**Table 2:** Intrapartum Outcomes

	Groups		
	
	Hypoglycemic (n = 436)	Controls (n = 434)	*P*-value
Cervical ripening	36 (8%)	38 (9%)	0.792
Induction of labor	129 (30%)	87 (20%)	<0.001
			
Delivery mode			
Spontaneous vaginal	314 (72%)	295 (68%)	
Assisted vaginal	15 (3%)	14 (3%)	
Cesarean section	107 (25%)	125 (29%)	0.364
			
Reason for cesarean section^(1)^			
Fetal distress	26 (24%)	23 (18%)	
Failure to progress	24 (22%)	36 (29%)	
Repeat cesarean section	35 (32%)	46 (37%)	
Breech/transverse lie	11 (10%)	9 (7%)	
Other	11 (10%)	11 (9%)	0.572
			
Reason for assisted vaginal delivery^(1)^			
Fetal distress	12 (80%)	10 (71%)	
Other	3 (20%)	4 (29%)	0.682
			
Fetal distress in labor	38 (9%)	33 (8%)	0.621
			
Cesarean delivery for fetal distress	26 (6%)	23 (5%)	0.769
			
Gestational age at delivery (med, min-max)	39 (23–41.5)	39 (28.6–41.6)	0.689
			
Gestational age distribution			
<37	67 (15%)	43 (10%)	
37–40	242 (56%)	273 (63%)	
>40	127 (29%)	118 (27%)	0.024
			
Gestational age at delivery <37	67 (15%)	43 (10%)	0.015

(1) Percentages for subsets of patients with condition present are shown.

Neonatal outcomes are shown in Table 3. Neonates born to women with hypoglycemia were of lower birth weight (median 3240 gm vs. median 3313 gm, p = 0.008). Similarly, the distribution of birth weight was different (p = 0.002), with a higher proportion of neonates with a birth weight less than 2500 gm in women with hypoglycemia. These differences, however, were not reflected in the incidence of IUGR which was similar in both groups (12% and 9% for hypoglycemic and control women, respectively, p = 0.277). Incidence of pH<7.1 and Apgar scores <7 at 5 minutes were similar between the groups (p = 0.130 and p = 0.374). No differences between admission to the NICU were found (p = 0.149).

**Table 3:** Neonatal Outcomes

	Group		
	
	Hypoglycemic (n = 436)	Controls (n = 434)	*P*-value
Birth weight (gm) (med, Q1-Q3)	3240 (2928–3550)	3313 (2980–3690)	0.008
Birth weight distribution			
<2500	43 (10%)	22 (5%)	
2500–4000	370 (85%)	370 (85%)	
>4000	23 (5%)	42 (10%)	0.002
IUGR	36 (12%)	29 (9%)	0.277
Umbilical artery pH<7.1	14 (3%)	7 (2%)	0.130
5 minutes Apgar score <7	4 (1%)	1 (.2%)	0.374
			
Admission to NICU	36 (8%)	25 (6%)	0.149
Admission reasons^(2)^			
Prematurity	6 (17%)	4 (16%)	
RDS/TTN	26 (72%)	12 (48%)	
Other^(2)^	4 (11%)	9 (36%)	0.054

(1) Medians and interquartile ranges (1^st ^quartile – 3^rd ^quartile) are shown; ^(2) ^percentages for subsets of patients with condition present are shown.

Three perinatal deaths occurred in the study, 2 in the hypoglycemic group and 1 in control women. The perinatal death in the control group was a neonate delivered at 36 weeks after prolonged rupture of the membranes that died in the NICU of sepsis. The first of the 2 deaths in the hypoglycemia group occurred following a 24 week delivery with the infant subsequently dying from complications of prematurity. The second death was a stillborn at 36 weeks secondary to a cord accident.

Multivariable analysis of predictors of hypoglycemia in pregnancy indicated age, pre-existing medical conditions and pre-pregnancy BMI categories (<19, 19–30, ≥30) as simultaneously significant risk factors. Age under 25 years (OR = 1.61, 95% CI 1.18–2.22, p = 0.003) and presence of pre-existing medical conditions (OR = 4.02, 95% CI 2.22–7.27, p < 0.001) increased the likelihood of hypoglycemia. Compared to women with pre-pregnancy BMI between 19 and 30, women with BMI ≥ 30 had a lower risk of hypoglycemia (OR = 0.60, 95% CI 0.42–0.87, p = 0.008), while women with BMI < 19 had similar likelihood of hypoglycemia in pregnancy (OR = 1.20, 95% CI 0.60–2.37, p = 0.608).

Evaluation of effects of hypoglycemia on pregnancy outcomes in multivariable logistic regression analyses indicated that, compared to women with 1 hour glucose between 89 and 139 mg/dL, women with hypoglycemia were more likely to develop preeclampsia/eclampsia (OR = 3.13, 95% CI 1.51–6.51, p = 0.002). Another simultaneous predictor of preeclampsia/eclampsia was presence of pre-existing hypertension (OR = 7.29, 95% CI 3.61–14.75, p < 0.001), while presence of other pre-existing medical conditions did not alter the risk (OR = 1.36, 95% CI 0.17–10.81, p = 0.769). Sensitivity of hypoglycemia in pregnancy for prediction of preeclampsia had 77.8% sensitivity, 51.4% specificity, 8% positive predictive power and 97.7% negative predictive power.

Hypoglycemia in pregnancy was not related to an indicated induction of labor (OR = 1.75, 95% CI 0.83–1.66, p = 0.361), once other risk factors were simultaneously considered including age under 25 (OR = 2.18, 95% CI 1.53–3.11, p < 0.001), presence of preeclampsia/eclampsia (OR = 11.63, 95% CI 5.23–25.85, p < 0.001), presence of pre-existing medical conditions (OR = 2.77, 95% CI 1.60–4.81, p < 0.001), and gestational age at delivery (p < 0.001).

Hypoglycemia in pregnancy appeared not to be related to the likelihood of preterm delivery (OR = 1.61, 95% CI 0.92–2.82, p = 0.093) when the adjustments for preeclampsia/eclampsia (OR = 9.05, 95% CI 4.28–19.16, p < 0.001) and preterm labor (OR = 39.34, 95% CI 16.31–94.87, p < 0.001) were made. No effects of hypoglycemia in pregnancy were associated with fetal distress in labor, cesarean delivery, IUGR, low pH, low Apgar scores at 5 minutes, or admissions to a NICU after adjusting for other risk factors (Table 4). No multivariable assessment of oligohydramnios was performed due to the small number of cases (n = 12).

**Table 4:** Adjusted odds ratios (OR) and the equivalent relative risks (RR) with their 95% confidence intervals (CI) that reflect the effects of hypoglycemia on select perinatal outcomes

Outcome	OR	95% CI	RR	95% CI	p-value
Preeclampsia	3.13	1.51–6.51	2.98	1.49–5.78	0.002
Induction of labor	1.75	0.83–1.66	1.52	0.86–1.47	0.361
Preterm delivery	1.61	0.92–2.82	1.52	0.93–2.39	0.093
IUGR	1.24	0.73–2.10	1.21	0.75–1.91	0.425
Macrosomia	0.49	0.28–0.85	0.51	0.30–0.86	0.011
Umbilical artery pH<7.1	2.00	0.76–5.26	1.97	0.77–4.93	0.159
5 min Apgar score <7	1.97	0.20–19.91	1.97	0.20–19.19	0.564
NICU admission	1.27	0.62–2.05	1.12	0.63–1.94	0.965

Preeclampsia: presence of pre-existing hypertension, presence of other medical history, maternal age.

Induction of labor: any pre-existing medical history, gestational age, preeclampsia, maternal age.

Preterm delivery: preeclampsia, antenatal hospitalizations, threatened preterm labor, labor induction.

IUGR: gestational age at delivery, pre-existing medical conditions present.

Macrosomia: maternal age, preeclampsia and gestational age at delivery.

Umbilical artery pH<7.1: gestational age at delivery and mode of delivery.

5 min Apgar < 7: gestational age at delivery and induction of labor.

NICU admission for any reason: gestational age at delivery, fetal distress in labor, cesarean delivery, IUGR, macrosomia.

## Discussion

Nearly universal screening of pregnant women is undertaken with a one hour GCT between 24 and 28 weeks of gestation except in women who are diabetic prior to the pregnancy or who have risk factors for diabetes and are screened at an earlier gestational age. Elevated blood glucoses on the one hour screen lead to further evaluation with a three hour glucose tolerance test. To date, an association of low blood glucoses to adverse pregnancy outcome remains uncertain. The literature that is present on hypoglycemia and pregnancy outcomes is old and a variety of glucose challenge tests have been used to define hypoglycemia in pregnancy. This study evaluated the predictability of a laboratory result that is widely available on pregnant women and its possible association with an unfavorable pregnancy outcome.

Conditions were identified by multivariate analysis in which the participants would be more likely to have hypoglycemia on the GCT. They include a maternal age < 25 years and the presence of preexisting medical conditions (chronic hypertension, chronic renal disease, thrombophilia, lupus, and seizure disorders). However, no difference in the risk of hypoglycemia was observed between women with a pre-pregnancy BMI of 19–30 vs. those with a BMI < 19, but as expected, women with a BMI > 30 had a significantly reduced incidence of hypoglycemia. This finding is consistent with the observation that women with a pre-pregnancy BMI > 30 are more likely to develop gestational diabetes and would therefore more likely have a blood glucose > 140 mg/dL (7.7 m/mol) on GCT [13].

The pregnancy effects of maternal hypoglycemia were then evaluated. Multivariate analysis revealed that women with hypoglycemia were more likely to develop preeclampsia/eclampsia. This result has not been reported before to our knowledge although in our pilot study the incidence of preeclampsia approached significance in those women with hypoglycemia following the GCT. We do acknowledge that the strength of this association is currently uncertain, as we have no precise information on the time that the glucose challenge test was undertaken and when the preeclampsia was diagnosed in each of the patients. The relationship was assumed based on an implicit close relationship in gestational age of the diagnosis of the hypoglycemia and the development of the preeclampsia. We can speculate that since preeclampsia is present early in pregnancy and alters placental implantation with a failure of vascular remodeling, the maternal/placental function is affected. Rather than an increasing resistance to insulin with the production of HPL by the placenta and other diabetogenic hormones and factors, as is observed in a normal pregnancy, these would be reduced resulting in less insulin resistance and a greater tendency for hypoglycemia following a glucose challenge test. The vasospasm of preeclampsia could also play some unknown role in altering the maternal response to a glucose load. Further investigations are needed to clarify if a relationship does exist between glucose loading and women who subsequently develop preeclampsia, and if so, the pathophysiology of that maternal response.

Indicated inductions of labor for a variety of reasons including rupture of membranes without labor, non reassuring antenatal testing, oligohydramnios, preeclampsia, intrauterine growth restriction and preterm premature rupture of the membranes were more commonly identified in the hypoglycemic group compared with women with a normal glucose following the GCT. As expected, this would suggest that women develop a variety of problems in the latter part of pregnancy that would result in an indicated induction. Hypoglycemia was also associated with a significant reduction in fetal macrosomia. This is not an unexpected finding as the hyperglycemia of gestational diabetes is linked with a greater risk of fetal macrosomia.

Also notable was the lack of association between hypoglycemia and preterm labor and/or delivery. The women with hypoglycemia were not at increased risk compared to women with a normal GCT for fetal distress in labor, cesarean delivery, low Apgar scores, low cord pH or admission to a neonatal intensive care unit. The lack of correlation between hypoglycemia and intrauterine growth retardation is different from the finding of most other investigators [1-7,9], except for 2 of the more recent studies which also evaluated hypoglycemia after the 50 gram oral glucose challenge test [8,11]. The older studies used a variety of techniques to identify the hypoglycemic pregnancies including higher both oral and IV glucose loading than that achieved with the oral 50-gram load as in our screening protocol. One possible explanation is that pregnancies identified as hypoglycemic by higher pre-test loads identify pregnancies in which the fetuses become growth restricted, but that the lower load with the 50-gram dose does not categorize the same women as hypoglycemic. Different patient demographics and different definitions of intrauterine growth restriction may also have contributed to the lack of significance with hypoglycemia in this study. Intrauterine growth restriction, low Apgar scores, umbilical artery pH and NICU admissions were all seen more frequently in the hypoglycemic group compared to the control group but the findings were not statistically significantly different. Even though this was a large prospective study with 870 women the possibility of a sample size error must be taken into consideration and that with larger number of participants a significant difference may be observed.

One of the weaknesses of this study may be that the glucose load to identify the pregnancies which are linked to an adverse pregnancy outcome may be too low. However, this must be balanced with the availability and ease of administration of the test. Use of the 50-gram load is very appealing to most pregnant women undergoing this test in the second trimester of pregnancy. The strength of this study is the large number of prospectively matched women which was evaluated and the multiple analyses undertaken to accurately determine which women are more likely to become hypoglycemic and the outcomes of those pregnancies.

Future investigations are needed to clearly describe the associations between women who are hypoglycemic following a glucose challenge, women with one abnormal value on a glucose tolerance test, and women with 2 abnormal values (gestational diabetics) with adverse pregnancy outcomes. The use of additional diagnostic tests such as the homeostasis model assessment (HOMA) with both insulin resistance (HOMO-IR) and beta cell function (HOMO-BETA) may well prove to be beneficial in further understanding of the links between maternal glucose and their effects on pregnancies. The observed association between hypoglycemia and oligohydramnios and the known link between hyperglycemia and hydramnios in addition to the witnessed relationship between oligohydramnios and intrauterine growth restriction and hydramnios with macrosomia provide a very fruitful area for further investigations to assist in the understanding of the role of maternal glucose and these connections.

## Conclusion

Women with hypoglycemia on GCT are younger and more likely to have a pre-existing medical condition prior to pregnancy. They may be more likely to develop preeclampsia during pregnancy but not intrauterine growth restriction.

## Abbreviations

GCT: glucose challenge test; GTT: glucose tolerance test.

## Competing interests

The authors declare that they have no competing interests.

## Authors' contributions

SBP and JBH collected the data on all the patients; DAD did all the statistical work for the paper; EFM helped collect data on patients, wrote the IRB and assisted on writing the paper. SPC and JCM helped with the planning and IRB submission and assisted in writing the manuscript.

## Acknowledgements

The views expressed in this article are the view of the author and do not necessarily reflect the official policy or position of the Department of the Navy, Department of Defense, or the US Government
